# Improving Infinium MethylationEPIC data processing: re-annotation of enhancers and long noncoding RNA genes and benchmarking of normalization methods

**DOI:** 10.1080/15592294.2022.2135201

**Published:** 2022-11-10

**Authors:** Martin Bizet, Matthieu Defrance, Emilie Calonne, Gianluca Bontempi, Christos Sotiriou, François Fuks, Jana Jeschke

**Affiliations:** a Laboratory of Cancer Epigenetics, Faculty of Medicine, Université Libre de Bruxelles (ULB), Brussels, Belgium; b Interuniversity Institute of Bioinformatics in Brussels (IB2), Université Libre de Bruxelles (ULB), Brussels, Belgium; c Institut Jules Bordet, ULB, Brussels, Belgium

**Keywords:** DNA methylation, 5mC, infinium, MethylationEPIC, EPIC, 850k, annotation, normalization, enhancers, long noncoding RNA

## Abstract

Illumina Infinium DNA Methylation (5mC) arrays are a popular technology for low-cost, high-throughput, genome-scale measurement of 5mC distribution, especially in cancer and other complex diseases. After the success of its HumanMethylation450 array (450k), Illumina released the MethylationEPIC array (850k) featuring increased coverage of enhancers. Despite the widespread use of 850k, analysis of the corresponding data remains suboptimal: it still relies mostly on Illumina’s default annotation, which underestimates enhancerss and long noncoding RNAs. Results: We have thus developed an approach, based on the ENCODE and LNCipedia databases, which greatly improves upon Illumina’s default annotation of enhancers and long noncoding transcripts. We compared the re-annotated 850k with both 450k and reduced-representation bisulphite sequencing (RRBS), another high-throughput 5mC profiling technology. We found 850k to cover at least three times as many enhancers and long noncoding RNAs as either 450k or RRBS. We further investigated the reproducibility of the three technologies, applying various normalization methods to the 850k data. Most of these methods reduced variability to a level below that of RRBS data. We then used 850k with our new annotation and normalization to profile 5mC changes in breast cancer biopsies. 850k highlighted aberrant enhancer methylation as the predominant feature, in agreement with previous reports. Our study provides an updated processing approach for 850k data, based on refined probe annotation and normalization, allowing for improved analysis of methylation at enhancers and long noncoding RNA genes. Our findings will help to further advance understanding of the DNA methylome in health and disease.

## Background

5-Methylcytosine (5mC) is an abundant epigenetic mark resulting from enzymatic addition of a methyl group (CH_3_) to the fifth carbon of a cytosine, typically in the context of a cytosine-guanine dinucleotide (CpG) in human DNA [1]. It has a central function in normal human development, as it controls gene transcription and other physiological processes such as chromosome stability, imprinting, and X-chromosome inactivation [1,2]. Several techniques have been developed to measure the genome-wide distribution of 5mC and to study its role in development and disease [3].

Whole-genome bisulphite conversion followed by sequencing (WGBS) offers single-base resolution and provides the most complete 5mC maps. Yet its high cost and the large amount of input material required limit its current use, especially in clinical studies of cancer and other complex diseases, which require profiling large patient cohorts from low amounts of input material [4]. For such studies, high-throughput technologies, such as reduced-representation bisulphite sequencing (RRBS) and Infinium HumanMethylation microarrays, are preferred [5–7]. RRBS uses a methylation-insensitive restriction enzyme to limit sequencing to regions of moderate to high CpG density [8]. Like WGBS, RRBS allows single-base resolution, but sample input and costs are lower thanks to reduced sequencing.

Illumina’s Infinium Human Methylation arrays assess methylation levels in large portions of the genome at single-base resolution by targeting selected cytosines. The Infinium HumanMethylation27 bead chip (27k) was released in 2008 and covers 27,578 CpGs primarily located within gene promoters [9]. The updated Infinium HumanMethylation450 bead chip (450k) covers more than 450,000 CpGs located within promoters, gene bodies, and intergenic regions [10,11]. Although this array shows probe-specific biases that need to be corrected [12], it has proven to be efficient and affordable for genome-scale differential methylation analysis. It was used, for example, by ‘The Cancer Genome Atlas’ (TCGA) [5] and by numerous other large-scale studies on cancer, diabetes, and ageing [13–18] .

High-throughput profiling of patient biopsies has highlighted perturbation of the DNA methylome as a hallmark of cancer. Numerous studies have shown 5mC alterations to contribute to the development and progression of breast and other cancers [19,20], demonstrating its potential as a cancer biomarker and cancer therapy target [21,22]. With technological progress in recent years, it has emerged that changes in the DNA methylome, under both physiological and pathological conditions, stretch beyond the regions (promoters and gene bodies) primarily covered by the 27k and 450k arrays [23–25]. In breast and other cancers, 5mC aberrations have been shown to occur most frequently at enhancers [26–29], and enhancer methylation appears to anti-correlate better than promoter methylation with target gene expression [3,30,31]. In addition, genomic regions transcribed to noncoding RNAs have been identified as frequent methylation targets. Among these, long noncoding RNAs (lncRNAs, defined as noncoding transcripts exceeding 200 nucleotides) have been shown to regulate key biological processes [32], and both their levels and 5mC patterns have been found altered in cancers [33,34].

With growing interest in enhancer methylation, Illumina released in 2016 the Infinium MethylationEPIC bead chip (850k), which targets more than 850,000 CpGs and features improved coverage of non-promoter regions, particularly enhancers. Although this array is widely used to study various diseases, analysis of its data remains suboptimal, as it relies mostly on the default probe annotation provided by Illumina [35–37]. According to this annotation, which identifies enhancers on the basis of the FANTOM5 database [38], 4.6% of the probes target enhancer-associated cytosines and 2.5% of the targeted cytosines are associated with noncoding RNAs [39]. As FANTOM5 enhancers were identified with the restrictive ‘Cap analysis gene expression’ (CAGE) method, the number of enhancers covered by the 850k array is grossly underestimated. Similarly, lncRNA genes are poorly mapped in Illumina’s default annotation.

To overcome these limitations, we have developed a novel approach based on the Encyclopaedia of DNA element (ENCODE) and LNCipedia databases to re-annotate 850k data. We have demonstrated the advantage of this approach over other technologies in several ways [1]: by comparing its coverage of regions corresponding to enhancers and lncRNAs with those of two other high-throughput technologies for profiling large patient cohorts (450k and RRBS) [2]; by using various normalization methods developed for 450k to investigate the reproducibility of the data obtained [3]; by using 850k with our new annotation to perform differential methylation analysis on breast cancer biopsies. Our study thus provides an updated analysis pipeline for 850k array data, based on refined probe annotation and normalization and allowing improved analysis of methylation at enhancers and lncRNA genes.

## Results

### Re-annotation of the 850k array

**Figure 1.**
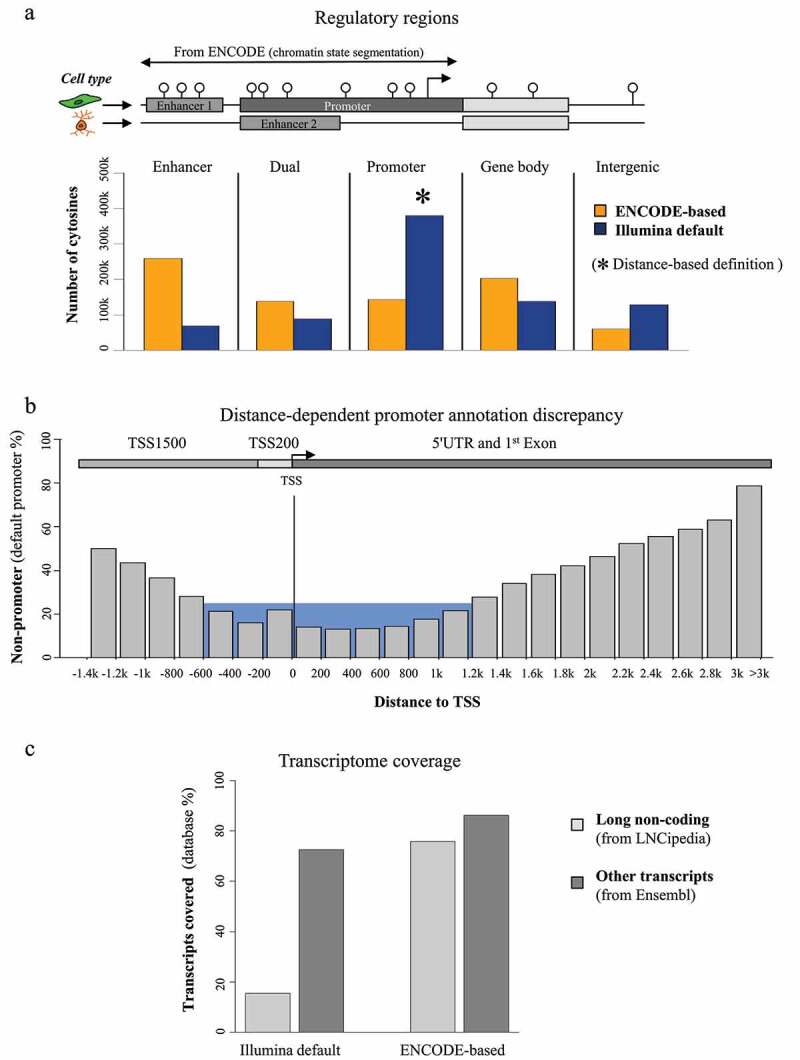
Reannotation of the 850k bead chip: (a) Barplot of the number of 850k-assessed cytosines associated with ‘enhancer,’ ‘dual,’ ‘promoter,’ ‘gene body,’ and ‘intergenic’ regions using ENCODE-based (orange) and Illumina default (blue) annotations The ENCODE-based annotation shows better coverage of all regulatory regions (‘Enhancer,’ ‘Dual,’ and ‘Gene Body’) except ‘Promoter’ regions (see the * mark), (probably because of an imprecise promoter definition in the Illumina default annotation). Top: lollipop scheme of the assessed regions. Cytosines are represented as white lollipops. The scheme shows two example cell types. Enhancers and promoters, identified through ENCODE chromatin state segmentation, are shown, respectively, in medium grey and dark grey. The gene body is represented in light grey. ‘Enhancer’ and ‘Promoter’ regions are defined as regions where only enhancers or promoters, respectively, can be identified through ENCODE chromatin states across all investigated cell lines. ‘Dual’ regions are associated with a promoter in some cell lines and with an enhancer in others. The TSS is represented as an arrow. (b) Barplot of percentages of probes associated with promoters by the Illumina annotation but not the ENCODE-based annotation as a function of distance to TSS. The grey bars represent percentages of probes showing a discrepancy between the Illumina and ENCODE-based annotations within a 200-bp window based on distance to the TSS. The blue region represents distance to TSS where the discrepancy is lower than 30%. The four promoter-associated regions (according to Sandoval et al [49]) are shown as a scheme at the top of the figure: TSS200 (light grey), TSS1500 (medium grey), 5’ UTR and 1st Exon (dark grey). The TSS position is specified by a vertical line in the barplot and as an arrow in the scheme. (c) Barplot of the percentage of transcripts associated with at least one 850k-targeted cytosine, according to the reference annotation (Illumina default left, ENCODE-based right) and transcriptomic database (LNCipedia light grey, Ensembl dark grey).

Illumina’s default annotation of the 850k array provides few clues for identifying enhancer-associated CpGs, even though enhancer coverage is one of its main features [39,40]. To overcome this limitation, we defined enhancers and other regulatory regions on the basis of the hidden Markov model provided by ENCODE (ENCODE-HMM) data (see GSE198627_mbizet_GEO_Methylation850k_PLATFORMv2.1.txt.gz on GEO). ENCODE-HMM provides enhancer locations based on chromatin immunoprecipitation followed by sequencing (ChIP-seq) of enhancer-specific histone marks such as H3K4me1 and H3K27ac. It features a larger number of enhancers than the restricted Functional annotation of mammalian genome version 5 (FANTOM5) database used by Illumina. With the ENCODE-based annotation, we were able to reduce the number of CpGs associated with ‘Intergenic’ regions by more than 50% (128,367 reduced to 60,236) and to improve the association of cytosines with ‘Dual’ regions 1.5-fold (88,822 to 137,952) and with ‘Enhancer’ regions more than 3.5-fold (68,509 to 259,096) (Figure 1a). Overall, our new annotation resulted in fewer non-annotated probes and increased the number of CpGs associated with enhancers. Importantly, using the EnhancerAtlas database, we were able to associate more than 120,000 enhancer-associated ENCODE CpGs with target transcripts. This means that more than 60% of the EnhancerAtlas target transcripts are associated with at least one CpG present on the 850k array. This essential information is missing from Illumina’s default annotation, but is now available through our ENCODE-based annotation as a supplementary file on GEO (GSE198627_mbizet_GEO_Methylation850k_PLATFORMv2.1.txt.gz).

Illumina’s annotation, which defines promoters based on distance from the TSS using the same four categories (i.e., TSS200, TSS1500, 1st Exon and 5'UTR) that were already used for 450k annotation, was found to associate more CpGs with promoter regions than the ENCODE-based annotation, which defines promoters through specific histone marks (Figure 1a). We found CpGs close to a transcription start site (TSS) to be consistently assigned to the ‘Promoter’ category in both the ENCODE-based and Illumina annotations, but regions outside the −600 to +1,200 range showed high discrepancy, increasing with the distance from the TSS and reaching more than 75% for regions located more than 3 kb from the TSS (Figure 1b). Overall, more than 25% of the CpGs annotated to the ‘Promoter’ category by Illumina were not confirmed by the ENCODE-based annotation, suggesting that Illumina’s annotation associates some CpGs with promoters incorrectly.

Next, to improve the annotation of 850k probes to noncoding transcripts, we used more than 80,000 lncRNAs from the LNCipedia database. In addition, we retrieved coding and small noncoding transcripts from the Ensembl database, so as to produce a global set of more than 250,000 transcripts [41,42]. In addition to the 120,000 CpGs we had already associated with transcription enhancement, we linked more than 300,000 CpGs to transcription initiation by assessing the overlap between a promoter region containing a targeted cytosine and the TSS corresponding to a transcript. Similarly, we identified more than 90,000 850k-targeted CpGs that might regulate enhancer RNAs (eRNA), a type of lncRNA subject to enhancer-controlled transcription initiation. Finally, more than 630,000 CpGs were associated with gene bodies, being located between the TSS and the transcription termination site (TTS) of a transcript-associated gene. As shown in Figure 1c, this approach allowed us to substantially improve coverage, particularly of lncRNA genes.

### Coverage of the re-annotated 850k array in comparison to 450k and RRBS

**Figure 2.**
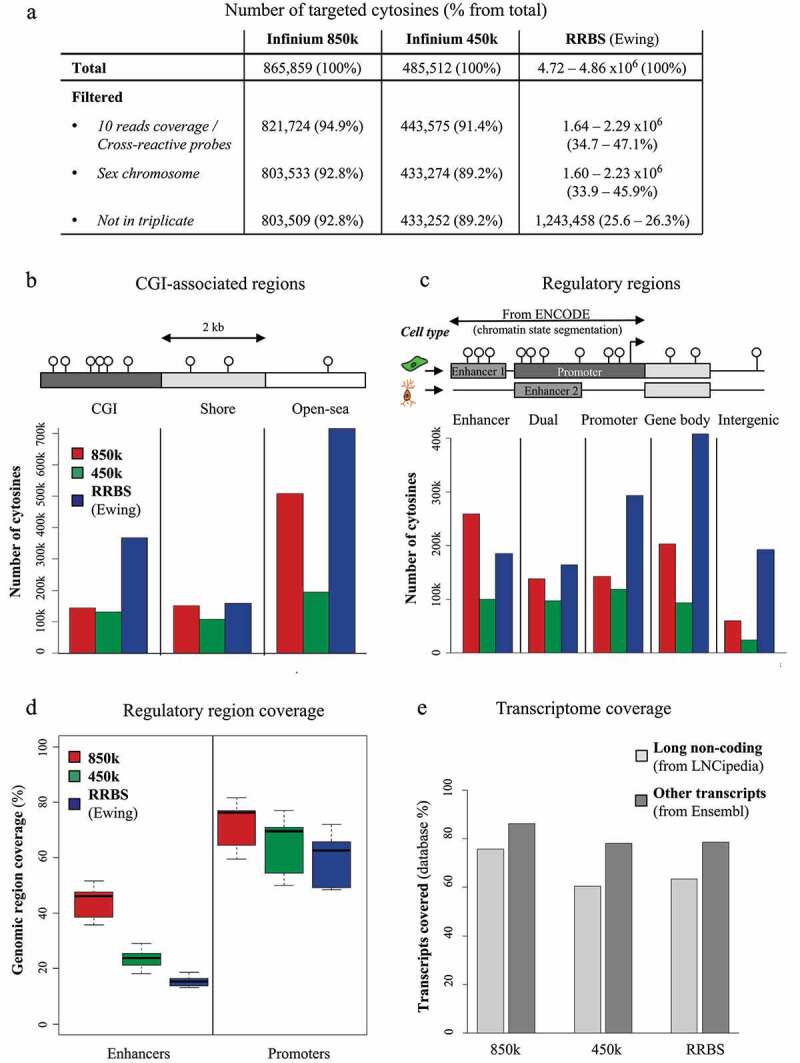
Coverage of the 850k bead chip as compared to 450k and RRBS: (a) Table showing the number of cytosines targeted by 850k, 450k, and RRBS through the filtering steps. Within parentheses: Percentages of the total number remaining after filtering. (b-c) Barplot of the number of cytosines covered by 850k (red), 450k (green), and RRBS (blue) according to the CGI-associated region (b) or regulatory region (c) where the cytosines are located. Top: lollipop scheme of the assessed regions. Cytosines are represented as white lollipops. (b) The scheme highlights that CGIs (dark grey) are CpG-dense regions, Shores (light grey) are regions located up to 2 kb from a CGI, and the Open sea (white) contains the remaining parts of the genome. (c) The scheme shows two example cell types. Enhancers and promoters, identified through ENCODE chromatin state segmentation, are shown, respectively, in medium grey and dark grey. A gene body (from Ensembl or LNCipedia databases) is represented in light grey. ‘Enhancer’ and ‘Promoter’ regions are defined as regions where only enhancers or promoters are identified, respectively, through use of ENCODE chromatin states across all investigated cell lines, while ‘Dual’ regions are associated with a promoter in some cell lines and with an enhancer in others. The TSS is represented as a black arrow. (d) Proportion of regulatory regions (enhancers left, promoters right) covered by at least one cytosine targeted by 850k (red), 450k (green), or RRBS (blue). Each boxplot represents the distribution of the coverage among the nine cell lines provided by ENCODE. (e) Barplot of the percentage of transcripts associated with at least one cytosine targeted by 850k (left), 450k (middle), or RRBS (right), according to the transcriptomic database (LNCipedia light grey, Ensembl dark grey).

We then compared the coverages of three high-throughput technologies suited for profiling large patient cohorts: the re-annotated 850k bead chip, its precursor 450k, and RRBS. For this, we used in-house data sets for HCT116 cells profiled with triplicate 450k and 850k arrays and a publicly available RRBS data set for Ewing sarcoma. Unreliable CpG measurements were filtered out, including cross-reactive probes (450k and 850k), low coverage reads (RRBS), CpGs located in the sex chromosomes, and CpGs not assessed in all three replicates. After filtering, the proportion of ‘reliable’ probes remained high for both 850k (92.8%) and 450k (89.2%). In contrast, only 25.6 to 26.3% of the CpGs assessed by RRBS remained after filtering, either because read coverage was insufficient (<10) or because the methylation level was not available for all three replicates (Figure 2a). Overall, we assessed, with the 850k array, almost 2 times as many as with the 450k array (803,509 *versus* 433,252 CpGs) and not quite two-thirds as many as with RRBS (803,509 *versus* 1,243,458 CpGs).

Next, we investigated the locations of the CpGs targeted by the three technologies. We first used the UCSC database (which Illumina also used to annotate their 850k platform) to map CpGs to ‘CpG Islands’ (CGI), ‘Shores’ (2 kb surrounding a CGI), or ‘Open sea.’ We found coverage of CpGs at CGIs to be higher with RRBS than with 450k or 850k (Figure 2b). Shore CpGs were similarly covered by all three technologies. Open-sea CpGs were covered mostly by RRBS, but coverage was much better with 850k (more than 500,000 covered) than with 450k. We then mapped the CpGs to ‘Enhancer,’ ‘Promoter,’ ‘Dual,’ ‘Gene body,’ and ‘Intergenic’ regions, using the same database, ENCODE-HMM, as for re-annotation of 850k. The 850k array showed better coverage of all ENCODE-HMM-derived categories than the 450k array, especially ‘Enhancer’ and ‘Gene body’ (Figure 2c). About 200,000 850k-targeted CpGs were assigned a ‘Gene body’ location (25% of the array). The ‘Enhancer’ category was the dominant one covered by the 850k array, since more than 250,000 of its probes were exclusively located in enhancers. If one adds to this the ‘Dual’ CpGs (nearly 140,000), almost half of the 850k array probes (49%) were found to target ‘enhancer CpGs.’ RRBS predominantly targeted CpGs exclusively assigned to ‘Promoter’ (24%), ‘Gene body’ (33%), or ‘Intergenic’ (15%) location, as compared to about 180,000 CpGs (15%) assigned to an ‘Enhancer’ location and 160,000 (13%) assigned to a ‘Dual’ location.

Using the ENCODE lists of regulatory regions for each cell line, we assessed in the 850k, RRBS, and 450k data the percentage of regulatory regions covered by at least one CpG. The 850k array outperformed both of the other technologies. It covered between 35.8 and 51.5% of the listed enhancers, according to the cell line assessed (Figure 2d), as compared to 18.3 to 29.1% for 450k and only 13.1% to 18.6% for RRBS. Interestingly, although RRBS featured more promoter-associated CpGs than the Infinium arrays (Figure 2b), it covered a lesser percentage of promoters (48.5 to 71.9%) than either 850k (between 59.5 and 81.7%) or 450k (50.1 to 77.0%) (Figure 2d). This is due to the fact that the promoters assessed by RRBS tended to have a higher CpG density: RRBS covered 25.7 to 47.1% of the promoters with at least 10 CpGs, as compared to 12.8 to 25.7% for 450k and 17.0 to 33.2% for 850k (Additional File 1: Fig. S1).

Finally, we compared the different technologies’ coverages of genomic regions corresponding to coding transcripts and lncRNAs. Surprisingly, RRBS covered only slightly more transcripts than 450k (63.4% vs 60.4% of the LNCipedia transcripts and 78.5% vs 78.2% of the Ensembl transcripts). In contrast, we were able to map 850k probes to 75.6% of the LNCipedia transcripts and 86.1% of the Ensembl transcripts (Figure 2e). Together, our results show that the 850k technology covers more promoters, enhancers, and transcribed sequences than 450k or RRBS.

### Normalization of the 850k array

Like the 450k array, 850k assesses methylated and unmethylated cytosine signals with either two beads emitting light in the same colour channel (Infinium I assay) or one bead emitting light in two different colour channels (Infinium II assay). As the use of two different assay types has been shown to introduce a bias into 450k array data, methods for correcting this bias have been developed [11,12]. We profiled with 850k arrays three replicates of the well-characterized HCT116 human colon cancer wild type (WT) cell line and of its double knock-out (DKO) derivative, in which DNA methylation is strongly reduced because of deletion of the *DNMT1* and *DNMT3B* DNA methyltransferase genes [43]. We plotted the beta-value densities separately for the Infinium I and Infinium II assays and observed, as previously shown for 450k data [11], a shift of the unmethylated and methylated signals between the Infinium I and Infinium II assays (Additional File 1: Fig. S2A). When we compared the between-replicate variabilities for each probe, we observed a greater variance for Infinium II probes than for Infinium I probes (median standard deviation = 0.034 for Infinium II vs 0.013 for Infinium I) (Additional File 1: Fig. S2B). Together, these results demonstrate that the use of two different assay types introduces the same bias into 850k data as previously observed with 450k data.

**Figure 3.**
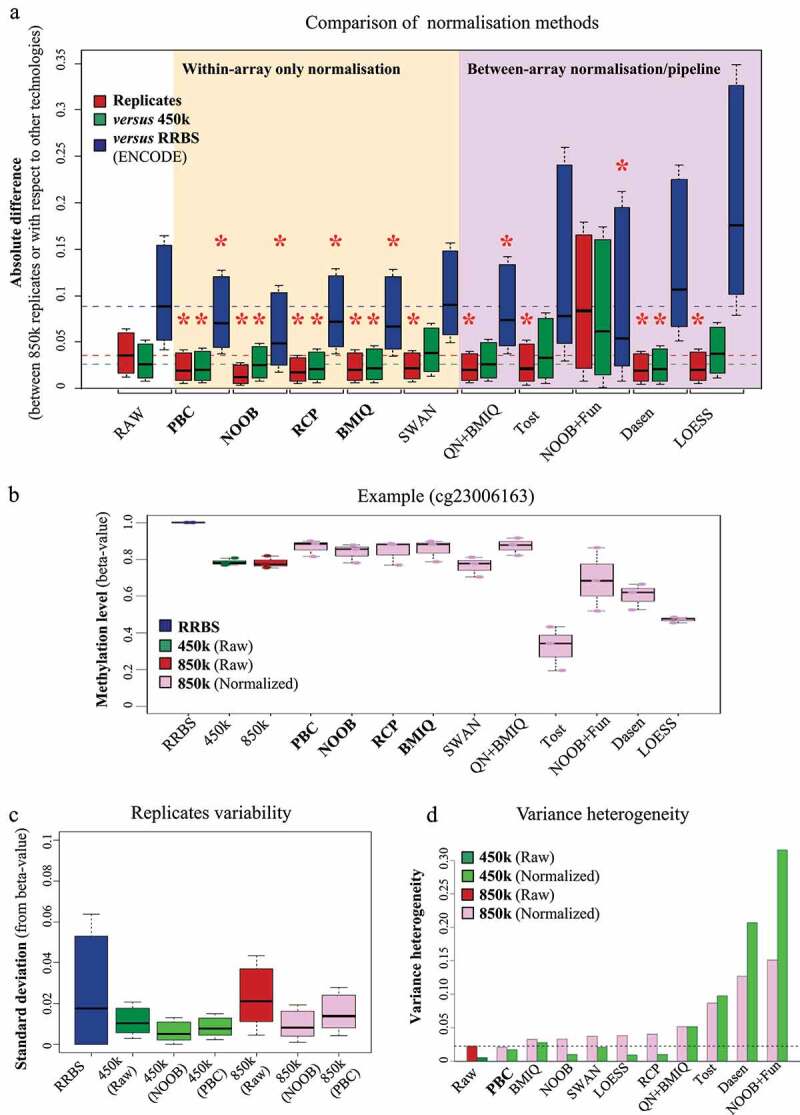
Evaluation of 850k-bias correction methods: (a) Boxplots showing the distribution of absolute differences between DNA methylation measurements obtained with Infinium 850k from three replicates of HCT116 WT cells (red) or between the 850k array and the 450k array (green) or between 850k and RBBS (blue), when the data are not normalized (white background), exclusively within-array normalized (orange background), or between-array normalized (possibly in a pipeline also including within-array normalization) (purple background). (b) Example of a probe impacted by the normalization method. The boxplots show the distribution of cytosine methylation levels assessed on RRBS duplicates (blue), 450k triplicates (raw measurement) (green), or 850k triplicates (raw measurement in red, normalized measurement in pink). The RRBS experiment shows a fully methylated cytosine but the raw 450k and 850k data show a beta-value lower than 1.0 . While some methods (e.g PBC, NOOB, and RCP) lead to data more similar to RRBS, others (e.g Dasen, LOESS) distort the data towards a hemi-methylated level. (c) Boxplots showing the distribution of the standard deviation obtained upon cytosine methylation level assessment with duplicate RRBS sequencing data (blue) or triplicate 850k-array (red) or 450k-array (green) measurements. The 450k and 850k data were subjected (dark-coloured) or not (light-coloured) to NOOB or PBC normalization. (d) Barplot showing levels of variance heterogeneity for HCT116 cell line methylation data: raw 450k data subjected (light green) or not (dark green) to normalization; 850k data subjected (pink) or not (dark red) to normalization. RAW: raw Infinium data; PBC: peak-based correction from the wateRmelon package; NOOB: Normal exponential convolution using out-of-bounds; RCP: regression on correlated probes method; SWAN: Subset quantile Within-Array Normalization from the minfi package; QN+BMIQ: pipeline formed by quantile normalization on intensities followed by beta-mixture quantile normalization; Tost: categorical SQN from the Touleimat and Tost pipeline; NOOB+Fun: pipeline composed of NOOB correction followed by functional normalization; Dasen: Dasen pipeline from the wateRmelon package. LOESS: local-regression between-array normalization.

To correct the observed bias of 850k, we applied the normalization methods developed for the 450k array. Adequate normalization strategies should both reduce between-replicate and between-technology variance, getting closer to the true value [12]. To evaluate the impact of normalization methods on intra- and inter-technology variance, we computed the absolute pairwise difference for each probe between each 850k replicate and i) the two other 850k replicates (Figure 3a, red), ii) the three replicates of 450k (green), and iii) the two replicates of RRBS (blue). As shown in the white box of Figure 3a, we observed in the absence of any correction (raw data) a lesser difference between replicates of 850k and 450k (median = 0.026) than between replicates of 850k and RRBS (median = 0.088). We then tested various within-array normalization methods (the ‘normal exponential convolution model using out-of-band intensities’ (NOOB) for correction of the background and the ‘peak-based correction’ (PBC), the ‘subset quantile for within-array normalization’ (SWAN), the ‘beta-mixture quantile dilation’ (BMIQ) and the ‘regression on correlated probes method’ (RCP) for normalization between type I and type II probes), several methods developed to simultaneously correct within- and between-array biases (the ‘quantile normalization on the intensity signal followed by BMIQ’ (QN+BMIQ), the preprocessing pipeline developed by Touleimat and Tost (Tost), the ‘NOOB followed by functional normalization pipeline’ (NOOB+Fun) and the ‘background correction and quantile normalization method treating types I and II separately’ (Dasen)), and a method developed to correct between-array bias only (local regression-based normalization (LOESS)). As shown in Figure 3a (within-array normalization methods in the yellow box and within/between-array normalization methods in the purple box), all the methods except NOOB+Fun reduced the variability between 850k replicates (intra-technology variance), but only PBC, NOOB, RCP, and BMIQ reduced differences between 850k and the two other technologies (inter-technology variance) (see Figure 3b for a specific probe).

We next compared the between-replicate variabilities of RRBS, 450k, and 850k (Figure 3c). We observed a higher median standard deviation between replicates of 850k than of 450k. This is likely due to differences in assay-type composition (Infinium I vs Infinium II) between the 450k and 850k arrays (Additional File 1: Fig. S2B, C). The median standard deviation was slightly higher for 850k raw data than for RRBS data, although some cytosines assessed by RRBS showed higher variability than in 850k. An in-depth analysis demonstrated that the standard deviation between RRBS replicates depends strongly on cytosine coverage. Hence, great sequencing depth is needed for reliable RRBS data (Additional File 1: Fig. S2D). Importantly, when the raw 850k data were corrected with NOOB or PBC, the between-replicate standard deviation decreased to a level comparable to that of corrected 450k data. Together, these findings clearly highlight the need to normalize 850k data prior to downstream bioinformatic analysis.

Statistical tests used to identify differentially methylated CpGs often require constant variability independent of the methylation level. Divergence from this property is called variance heterogeneity. To evaluate the variance heterogeneity of 850k data, we computed the distance between the mean standard deviation, representing the expected profile in a homogeneous variance context, and a local regression model of the standard deviation along the methylation profile, reflecting the observed situation (Additional File 1: Fig. S2E, F). This analysis revealed higher variance heterogeneity for 850k than for 450k raw data (Figure 3d red vs green). Notably, the normalization methods developed for 450k did not efficiently correct the variance heterogeneity of the 850k data (Figure 3d, pink rectangles). Some methods (NOOB and SWAN) increased variance at unmethylated sites (Additional File 1: Fig. S2E), others (Dasen or Tost) at methylated sites (Additional File 1: Fig. S2F). PBC was the only method found to reduce variance heterogeneity along the entire profile (Additional File 1: Fig. S2E) as well as at the global level compared to 850k raw data (Figure 3d dashed line). All other correction methods increased variance heterogeneity of both 850k and 450k data but for most of them (i.e., PBC, BMIQ, NOOB, SWAN, LOESS, and RCP), variance heterogeneity remained higher for the corrected 850k than for the corrected 450k data (Figure 3d light green *vs* pink). We therefore recommend that 850k users employ a statistical approach less sensitive to variance heterogeneity (*e.g.*, a non-parametric test or Welch’s version of the t-test) and that they be cautious of a higher risk of false-positive results. We did not investigate RRBS data, as their non-continuous nature allows the use of statistical tests that do not require variance homogeneity.

### Differential methylation analysis with 850k

Finally, we used the 850k array to perform differential methylation analyses. In addition to HCT116 DKO and HCT116 WT cells, we profiled four fresh-frozen biopsies from normal human breast tissue and ten from human breast tumours. The raw 850k data were normalized by PBC, because this was the only method shown, in our previous tests, to improve both data reproducibility and variance heterogeneity. We then compared the methylation profiles of HCT116 DKO vs HCT116 WT cells and primary breast cancer tissues vs normal breast tissues.

The 850k profiles of HCT116 DKO and HCT116 WT cells revealed 336,934 differentially methylated cytosines covering 41.9% of the array, 97.5% of which were hypomethylated in HCT116 DKO cells (Additional File 1: Fig. S3A). The differentially methylated probes covered all regulatory-region categories. They included both CpGs common to 850k and 450k and CpGs specific to 850k (Additional File 1: Fig. S3B), and it would seem that the CpGs covered only by 850k can reflect 5mC changes as efficiently as the CpGs already targeted by the 450k array. Striking differences in annotation of the differentially methylated probes were observed according to whether we used our ENCODE-based or the default Illumina approach. ENCODE-based annotation identified aberrant DNA methylation mostly in enhancers, whereas Illumina’s annotation highlighted promoters as most affected.

**Figure 4.**
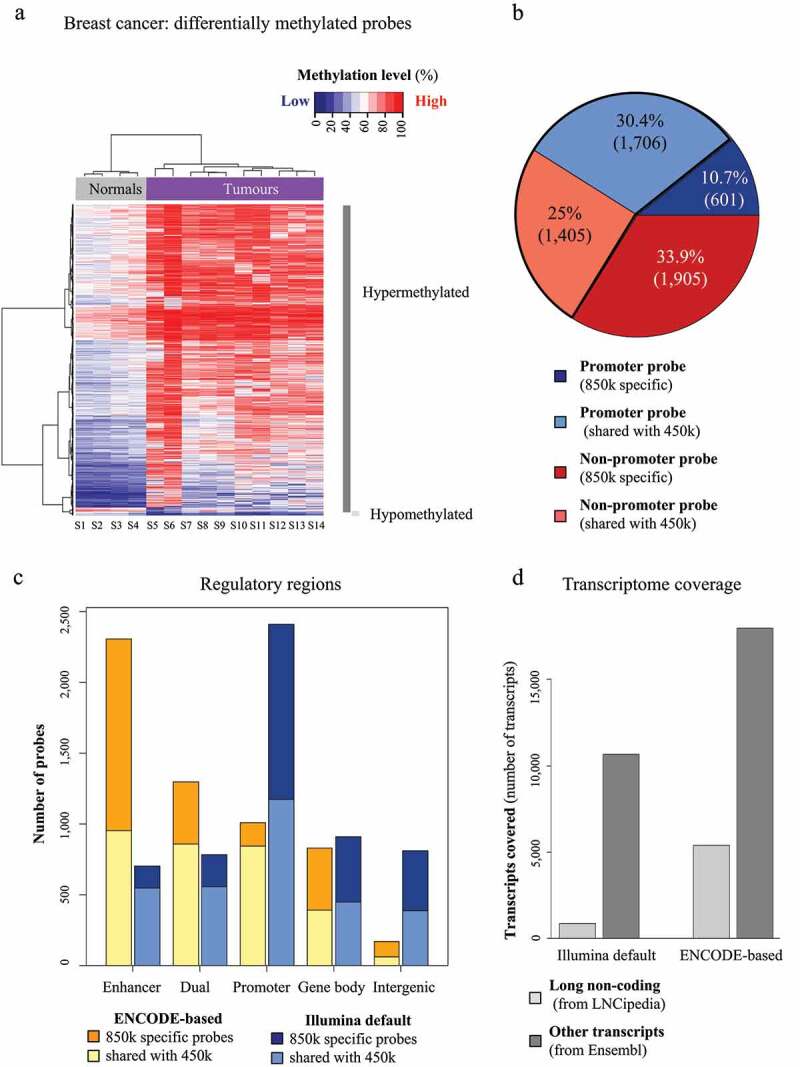
Differential methylation analysis of breast tumours vs normal samples with 850k: (a) Heatmap of the differentially methylated probes identified on the 850k array upon comparing breast tumour samples (samples S5 to S14, purple rectangle) with normal breast tissue samples (samples S1 to S4, grey rectangle) after PBC normalization. The methylation level is represented on a blue (unmethylated) to red (methylated) scale. Hypermethylated and hypomethylated probes are highlighted respectively a dark grey and a light grey vertical bar. (b) Pie chart of the proportion of differentially methylated promoter and non-promoter probes. Blue: promoter, red: non-promoter, light: probes common to the 850k and 450k versions, dark: probes specific to the 850k array. The part surrounded in black contains probes common to the two versions. (c) Barplot of the number of differentially methylated cytosines that can be assessed exclusively by 850k (dark) or that are common to 850k and 450k (light). The differentially methylated cytosines are associated with ‘Enhancer,’ ‘Dual,’ ‘Promoter,’ ‘Gene body,’ and ‘Intergenic’ regions according to the ENCODE-based (orange) or Illumina default annotation (blue). The ENCODE-based annotation shows a lower proportion of differentially methylated cytosines not associated with any feature (i.e., ‘Intergenic’). This leads to better interpretability of results. (d) Barplot showing the number of transcripts associated with at least one differentially methylated cytosine, according to the reference annotation (Illumina default left, ENCODE-based right) and the transcriptomic database (LNCipedia light grey, Ensembl dark grey). It highlights strong improvement in the number of transcripts identified with the ENCODE-based annotation as compared to Illumina’s default annotation.

**Figure 5.**
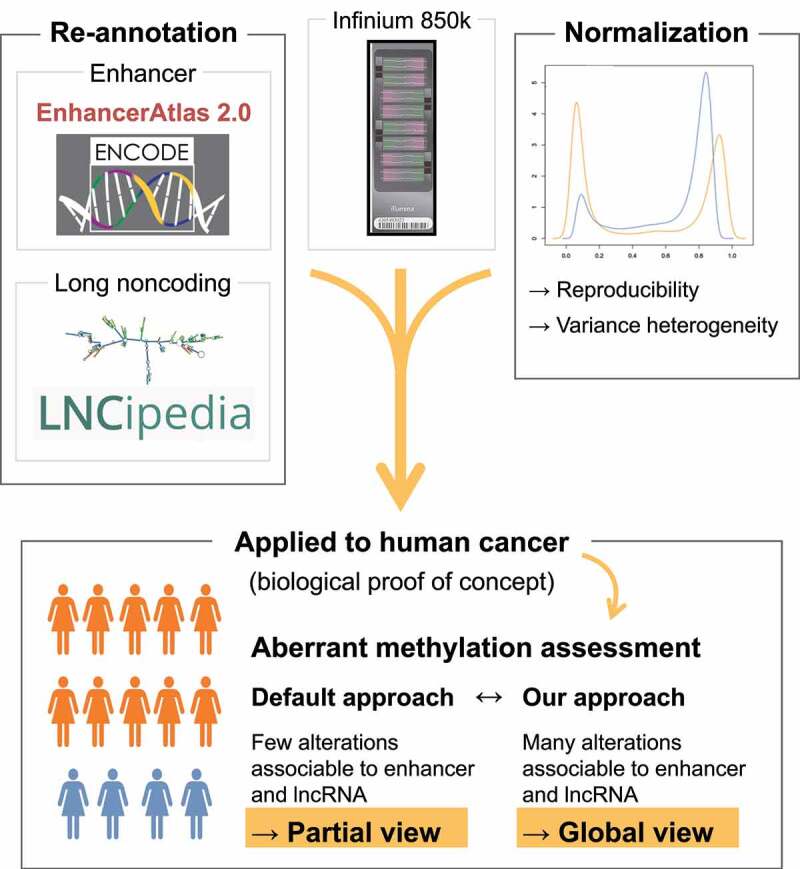
An updated processing approach for 850k data: Our new processing approach for 850k data, based on refined probe annotation and normalization, allows for improved analysis of DNA methylation at enhancers and long noncoding RNA genes. This approach highlights, as previously reported, aberrant enhancer methylation as a dominant feature in breast cancer. Thus, the 850k array, together with our new processing approach, allows for improved high-throughput, low-cost analysis of DNA methylation in clinical samples.

Comparison of the 850k profiles of primary breast cancer and normal breast tissues highlighted 5,617 differentially methylated CpGs, the majority of which were hypermethylated in tumours (Figure 4a). Of the differentially methylated CpGs, 33.9% (1,905 probes) were associated with 850k-specific non-promoter sites and would thus have been missed with the 450k array (Figure 4b). Again, we observed striking differences in annotation according to the approach used. With Illumina’s default annotation, alterations appeared mainly in promoter regions (Figure 4c). This is likely due to over-estimation of promoters by this annotation. The ENCODE-based annotation revealed differential methylation mainly in enhancers (particularly of 850k-specific CpGs). Lastly, we found our annotation to associate more transcripts (6 times as many lncRNA transcripts [5,416 *versus* 900] and 1.7 times as many other transcripts [17,969 *versus* 10,656]) with differentially methylated CpGs than Illumina’s default annotation (Figure 4d). We obtained similar results with BMIQ, another normalization tool that efficiently improved data reproducibility and only slightly increased variance heterogeneity (Additional File 1: Fig. S3C to F). Together, these findings suggest that our new 850k processing approach, based on refined probe annotation and normalization, identifies aberrant enhancer methylation as a dominant feature in breast cancer by allowing for a more global view into the DNA methylome of breast cancer (Figure 5).

## Discussion

Illumina’s 850k array has become popular for efficient, affordable genome-scale differential methylation analysis on large patient cohorts [44–46]. Shortly after its release, Moran et al. (2016) validated its performance, demonstrating high consensus between 850k and 450k data for technical replicates and for fresh-frozen and formalin-fixed paraffin-embedded samples [39]. A major drawback of this study is that it was based on Illumina’s default probe annotation, which underestimates the number of enhancers covered by the 850k array because it relies on enhancers identified by the restrictive CAGE method (FANTOM5 database). Surprisingly, up to now most studies still rely on Illumina’s default probe annotation, which is suboptimal as it grossly underestimates the number of enhancers and long noncoding RNAs. To overcome this limitation, we have developed an alternative probe annotation for the 850k array, based on the enhancers defined by ENCODE, identified through a less restrictive approach: ChIP of enhancer-specific histone marks such as H3K4me1 and H3K27ac [47,48]. Using ENCODE has enabled us to increase more than three-fold the number of enhancer-associated 850k array probes and thus to improve the power of this array to study dysregulation of enhancer methylation. Unlike Zhou et al. (2017), who also proposed a basic association between 850k probes and ENCODE chromatin states [40], our 850k re-annotation provides both cell-line-specific categories and a summary of categories for each probe, greatly improving the interpretability of data. More importantly, we have used EnhancerAtlas to associate target transcripts with a large proportion of the enhancers covered by 850k, making it possible to associate methylation changes at enhancers with genes and biological processes.

Our new annotation has also improved the association of probes with promoters. Investigators are still striving to provide a uniform, meaningful, genomic-distance-based definition of a promoter, and previous studies have applied different definitions. Illumina’s default annotation provides four categories that can be associated with promoters: ‘TSS 1500,’ ‘TSS 200,’ ‘5’ UTR’, and ‘1st Exon.’ Sandoval et al. (2011) merged these four categories into a single promoter region [49]. Dedeurwaerder et al. (2011) used an alternative, more restrictive definition, including only the ‘TSS 1500’ and ‘TSS 200’ categories and completely ignoring the intragenic part of the promoter [11]. Here, we have used the ENCODE HMM-based definition of promoters, because a promoter defined experimentally on the basis of histone marks is expected to be closer to the ‘true’ biological promoter [50]. A comparison of our ENCODE-based promoters with genomic-distance-based promoters suggests that the definitions based on Illumina’s default categories, as proposed by both Sandoval et al. and Dedeurwaerder et al., are too permissive, particularly for CpGs located further away from a TSS. We reveal a range from −600 to +1200 surrounding the TSS, within which discrepancies between the distance-based and ENCODE-based promoter definitions are low. We suggest that this range should be used as promoter definition when only genomic distance is available.

As DNA methylation changes corresponding to noncoding RNAs, particularly lncRNAs, can affect key cancer pathways [33,34,51], we have aimed to improve annotation of 850k probes to noncoding transcripts. Using the LNCipedia database, we have tripled the number of lncRNAs linked to 850k probes. Notably, among the transcripts corresponding to 850k probes, we have identified more than 90,000 eRNAs, a type of lncRNA whose synthesis is controlled by enhancers and which is thought to be important in enhancer-promoter looping [52]. In addition, using an updated transcriptome version from Ensembl (v93), we have increased by 10% the number of coding-transcript-associated 850k probes. This has enabled us to multiply by 1.6 the number of coding transcripts identified as being differentially methylated between normal and breast cancer tissue.

With assessment of more than 800,000 CpGs, the 850k array covers about 3% of all CpGs in the human genome (more than 28 million). Coverage by RRBS ranges from 1.8 to 71.4%, depending on the sequencing depth [53]. The publicly available ‘RRBS Ewing’ data set used in this study includes methylation values for 4.8 million CpGs, i.e., six times as many CpGs as the 850k data. Yet measuring methylation with RRBS has several limitations. First, as methylation value precision depends on sequencing depth, it is recommended to filter out low-coverage CpGs. Here we have followed ENCODE recommendations, keeping only CpGs covered by at least 10 reads [54]. This reduces the number of targeted CpGs by 50 to 60%. Secondly, we show that RRBS suffers from high sequence-coverage-dependent between-replicate variability and from inconsistent coverage of CpGs between experiments. In the ‘RRBS Ewing’ data, out of 1.6 to 2.29 million sites having survived read-coverage and sex chromosome filtering, only 1.2 million were covered in triplicate. In the 850k data, in contrast, only 24 CpGs were not available in triplicate. Altogether, the ‘RRBS Ewing’ data covered 1.55 times as many CpGs as the 850k array.

Our analysis further reveals differences in the types of regulatory genomic regions covered by the three technologies. As compared to 450k, the 850k array shows improved coverage of all ENCODE-HMM-derived categories, especially the ‘Enhancer’ and ‘Gene body’ categories. Enhancer-located CpGs are in fact the dominant feature of 850k (46% of all probes). RRBS, on the other hand, predominantly covers CpGs associated with CGIs, promoters, and gene bodies. Overall, 850k targets twice as many enhancers as 450k and three times as many as RRBS (cf. Figure 2d). Surprisingly, 850k also targets more promoters than either 450k or RRBS. Yet when interpreting 850k profiles, one must keep in mind that many regulatory regions are covered by only one CpG. RRBS targets fewer promoters and enhancers than 850k (or even 450k), but with several CpGs per region. The two technologies thus provide slightly different pictures of the methylome: RRBS assesses fewer regions but with several CpGs per region, while 850k provides a broader view at the cost of fewer CpGs per region. It is important to consider this difference when assessing methylation at promoters and in distal regulatory elements (e.g., enhancers), as methylation can vary across a region [55]. Pidsley et al. have shown that a single 850k probe is not always informative for distal regulatory elements with variable methylation [56]. Yet this same study demonstrated that more than 80% of the distal regulatory elements targeted by a single 850k probe accurately represented DNA methylation across the entire region. Thus, teams aiming to investigate regulatory regions with 850k are advised to use an independent technology to interrogate or validate methylation patterns across critical regions of interest.

As previously shown for the 450k array [12], the 850k array generates biases, as it also relies on two different probe types (Infinium I and II). The 850k-specific probes are primarily Infinium II probes, which are less accurate, less reproducible, and considerably less sensitive for detection of extreme methylation values than Infinium I probes. Although various tools exist to normalize this bias in 450k data, it remains to be evaluated whether they work efficiently in 850k data. Indeed, many of these tools rely for normalization on the probe composition, which is different between 450k and 850k arrays. Using normalization methods developed for the 450k array, we have succeeded in minimizing 850k biases. Among the within-array normalization strategies examined, methods correcting for differences in methylation distribution modes between Infinium I and II probes (PBC, BMIQ and RCP [11,57,58]) and NOOB, a method using ‘out-of-bounds’ intensities as negative controls to correct for background [59], improved 850k data reproducibility both within and across technologies, as demonstrated previously on 450k data [12,60]. In our data set, methods separating data into subgroups to normalize them independently (SWAN, Tost and Dasen [61–63]) improved 850k data reproducibility within but not across technologies, likely because they depend strongly on the validity of the defined subgroups. This shows a potential systematic bias of these normalization methods and highlights the necessity of our approach to always compare to another technology when evaluating a normalization method [12]. It is worth stressing that some normalization methods work on the assumption that most of the array does not change. The hypotheses underlying such methods can be invalid when major changes in methylation are investigated. For instance, HCT116 DKO cells are, by design, strongly depleted of methylated CpGs. Also, highly abnormal methylation patterns can arise through pharmacological DNMT1 inhibition [21] or alteration of the methylation machinery, as observed in cancer [64]. It is important to be aware of the risk of bias. On the basis of our comparison of different normalization methods, we advise 850k users to apply within-array normalization and preferably one of the four methods that improved reproducibility between technologies: PBC, RCP, NOOB and BMIQ. As variation between arrays is increased in larger cohorts [65], between-array normalization may be considered, but one should always compare the before- and after-normalization methylation density profiles to avoid data distortion. Moreover, variance heterogeneity, which is very low and neglectable in 450k data, should be taken into account for the analysis of 850k data where it is much higher. Noteably, among the four methods that improved data reproducibility, PBC was the only one shown to reduce variance heterogeneity although the level remained above that of raw or corrected 450k data. It is therefore essential to use statistics that are robust to variance heterogeneity (*e.g.*, a non-parametric test or Welch’s version of the t-test) and to be cautious of a higher risk of false-positive results when working with 850k data.

We have further used the 850k array to characterize breast cancer methylomes. The majority of differentially methylated CpGs were detected with 850k-specific probes and would have been missed with the 450k array. On the basis of our new annotation, we have found the largest proportion of 850k-specific probes showing changes in methylation to be associated with non-promoter regions, specifically enhancers. This finding is in agreement with sequencing-based observations on breast and other cancers [66–68]. It contrasts with the results obtained with Illumina’s default annotation, which highlighted promoters as the most affected category (cf. Figure 4c). Use of our new annotation also led to a substantial increase in the number of noncoding transcripts found to undergo changes in DNA methylation in breast cancer. Indeed, 850k used with the new annotation identifies aberrant enhancer methylation as a dominant feature in breast cancer, but at lower cost, with less input material, and with a higher throughput than WGBS. The 850k array, if its data are analysed with our improved pipeline based on re-annotation and normalization, is thus the technology best suited for investigating changes in DNA methylation in regulatory regions (enhancers, promoters, lncRNA genes) and coding regions in diseases requiring profiling of large patient cohorts.

## Conclusions

We here propose an alternative annotation for Illumina’s 850k array, associating its probes with promoters and enhancers identified by ENCODE. We have also used two complementary databases (Ensembl and LNCipedia) to improve association of 850k probes with both coding and noncoding transcripts. Our annotation results in fewer non-annotated probes than Illumina’s default annotation and in a higher number of enhancer-associated probes. Even though the total number of CpGs analysed with the 850k array is lower than the number analysed with RRBS, its CpGs are distributed over more regulatory regions, especially enhancers and promoters. This makes the 850k array the technology of choice for studying methylation changes in diseases requiring profiling of large patient cohorts. Furthermore, normalization improves both the between-replicate and between-technology reproducibility of 850k data. Lastly, with our new annotation, we identify aberrant enhancer methylation as a dominant feature in breast cancer, as reported in the literature. In conclusion, our findings suggest that the 850k array, together with our new annotation, allows improved high-throughput, low-cost analysis of DNA methylation at promoters, enhancers, lncRNA genes, and coding regions.

## Methods

### Samples and DNA extraction

Wild-type and double-knockout HCT116 cells (respectively called HCT116 WT and HCT116 DKO) were cultured in triplicate in McCoy’s 5A medium supplemented with 10% foetal calf serum at 37°C under 5% CO2. Genomic DNA was extracted with the QIAamp DNA Mini Kit (Qiagen, Hilden, Germany) and with the recommended proteinase K and RNase A digestions. Ten fresh-frozen breast tumour samples and four normal breast tissue samples, previously profiled with the 450k array [69], were obtained from patients diagnosed with breast cancer at the Jules Bordet Institute between 1995 and 2003, following approval by the Medical Ethics Committee of Institute Jules Bordet, Brussels, Belgium. All patients gave written informed consent before their participation in the study. Genomic DNA from frozen samples was extracted from 10-μm sections with the Qiagen DNeasy Blood and Tissue Kit according to the supplier’s instructions (Qiagen). The procedure included proteinase K digestion at 55°C overnight. DNA was quantified with the NanoDrop® ND-1000 UV–Vis Spectrophotometer (NanoDrop Technologies, Wilmington, DE, USA).

### Bisulphite conversion and DNA methylation profiling with the 450k and 850k arrays

Genomic DNA (800 ng) treatment with sodium bisulphite was done with the Zymo EZ DNA Methylation KitTM (Zymo Research, Orange, CA, USA) according to the manufacturer’s procedure, with the alternative incubation conditions recommended when using the Illumina Infinium Methylation Assay. The methylation assay was performed on 4 μl bisulphite-converted genomic DNA at 50 ng/μl according to the Infinium HD Methylation Assay protocol. Bead chip arrays were scanned on Illumina Scan and raw .idat files were generated.

### Array data analysis

All analyses were done with R (v3.4.4) except CpG annotation, which was done with python (v2.7.15).

## Data loading

Depending on the normalization method, two different loading methods were used. The read.metharray function of the minfi bioconductor R package (v1.24.0) was used to load data into a minfi-specific RGChannelSet object when normalization methods requiring this object were used. For the other normalization methods, raw .idat files of 450k data were loaded with the methylumi bioconductor R package (v2.24.1), while 850k data were loaded with the illuminaio package (v0.20.0). The detection p-values were obtained with Genome Studio® software (v1.6) and probes with detection p-values ≥ 0.05 were filtered out. Data quality was checked visually with control probes and all samples passed this quality control.

## Data normalization

All data normalization methods were applied usig one of the following five procedures:

i) Peak-based correction (PBC) [11], the preprocessing pipeline developed by Touleimat and Tost (Tost) [61], and the background correction and quantile normalization method [62] treating types I and II separately (Dasen) were run with the wateRmelon package and array-specific annotation files provided by Illumina (‘MethylationEPIC_v-1-0_B4.csv’: http://emea.support.illumina.com/downloads/infinium-methylationepic-v1-0-product-files.html and ‘HumanMethylation450_15017482_v.1.2.csv’: http://emea.support.illumina.com/array/array_kits/infinium_humanmethylation450_beadchip_kit/downloads.html).

ii) The normal exponential convolution model using out-of-band intensities (NOOB) [59], the subset quantile for within-array normalization (SWAN) [63], and the NOOB followed by functional normalization pipeline (NOOB+Fun) [65] were run with the appropriate functions of the minfi package (*i.e.*, preprocessSWAN, preprocessNOOB and preprocessFunnorm, respectively).

iii) The regression on correlated probes method (RCP) [57] was run with the method of the ENmix bioconductor R package (v1.14) after applying the preprocessRaw method of the minfi package.

iv) The pipeline proposed by Marabita et al [60]., consisting in quantile normalization on the intensity signal followed by beta-mixture quantile dilation (QN+BMIQ), was run with the lumiMethyN function of the lumi package and the BMIQ function (v 1.1) (code available at https://code.google.com/archive/p/bmiq/) while BMIQ was run with this last function alone.

v) LOESS between-array normalization from Heiss et al [70]. was adapted from the R code provided with the associated paper.

## Probe filtering

After normalization, ambiguous probes from both sex chromosomes were discarded. Cross-reactive probes identified by Price et al [71]. were filtered out of the 450k data and the annotation of McCartney et al [72]. was used to filter out cross-reactive 850k probes. Breast cancer data were also filtered against probes targeting SNPs identified by Price et al. (450k) or McCartney et al. (850k). Raw data (.idat) and preprocessed data were submitted to the Gene Expression Omnibus public database (GEO) (www.ncbi.nlm.nih.gov/geo/) (GSE198628).

### Reduced-representation bisulphite sequencing (RRBS) data

Two already-processed public RRBS datasets were used with different aims:

i) For CpG coverage comparisons, processed RRBS data for three Ewing sarcoma samples were downloaded from GEO (https://www.ncbi.nlm.nih.gov/geo/query/acc.cgi?acc=GSE89026) [73]. CpGs located in both sex chromosomes or covered by less than 10 reads were filtered out. Only CpGs whose methylation status was available for all three samples were analysed.

ii) For the comparison of methylation levels, RRBS data on HCT116 cell samples from two independent experiments were downloaded from ENCODE (https://www.encodeproject.org/experiments/ENCSR000DFS/). To simplify the comparison with bead-array technology, strand specificity was not taken into account. When methylation values were available for both strands of the same CpG site, the values were averaged if the two values were similar (delta < 10%) and discarded otherwise. Only cytosines common to the 450k and 850k arrays and covered by at least 10 reads were kept for subsequent analysis. In order to maximize the number of methylation-level comparisons between 850k and RRBS, cytosines whose methylation levels were available for only one sample were kept in this dataset.

### CpG annotation

CpGs were annotated with human genome build hg19. Ensembl positions were lifted from hg38 to hg19 with the ‘LiftOver’ tool from UCSC (https://genome.ucsc.edu/cgi-bin/hgLiftOver).

## CpG islands

CpG island (CGI) positions were retrieved from the UCSC database (https://genome.ucsc.edu/cgi-bin/hgTables). CGI shores were generated by adding 2 kb at both ends of each CGI. 850k, 450k, and RRBS data were overlapped with CGIs and Shores, using a custom python script. CpGs not located in a CGI or a Shore region were annotated as ‘Open-sea.’

## Regulatory regions

Regulatory genomic regions were retrieved with the ‘UCSC ENCODE Experiment Matrix tool’ (https://genome.ucsc.edu/ENCODE/dataMatrix/encodeDataMatrixHuman.html) for all available cell lines (HMEC, HSMM, K562, NHEK, NHLF, HEPG2, HUVEC, HESC, and GM12878). For each cell line, the hidden Markov model provided by ENCODE classifies the whole genome into 15 chromatin states. In order to simplify this annotation, we grouped the ‘1_Active_Promoter,’ ‘2_Weak_Promoter,’ and ‘3_Poised_Promoter’ states into a ‘Promoter’ super-region while ‘4_Strong_Enhancer,’ ‘5_Strong_Enhancer,’ ‘6_Weak_Enhancer’ and ‘7_Weak_Enhancer’ states were assigned to the ‘Enhancer’ super-region. The other states were ignored. States from the same super-region succeeding each other in the genome were fused. RRBS, 850k and 450k data were then overlapped with the ‘Promoter’ and ‘Enhancer’ super-regions in each cell line. As a CpG can be associated with a ‘Promoter’ super-region in one cell line and an ‘Enhancer’ in another, we assigned such CpGs to a third category called ‘Dual’ to reflect their dual role.

## Transcripts

Coding and noncoding transcript positions were retrieved from the LNCipedia high-confidence set version 5.2 and from Ensembl v93. Duplicates between the two databases were identified with the ‘lncipedia_5_2_hc_hg19.gff’ file from LNCipedia and only the LNCipedia version of each duplicated lncRNA was kept. Several CpG-to-transcript association types were investigated:

i) Association with the transcription start site (TSS): each transcript whose TSS was located in a ‘Promoter’ super-region was assigned to that promoter and associated with the CpGs of that promoter. If a TSS for an lncRNA transcript fell into an ‘Enhancer’ super-region, the transcript was associated with that region and defined as an eRNA (enhancer RNA). The list of ‘Promoter’/‘Enhancer’-transcript associations is available in Table S1 (Additional File 2).

ii) Enhancer targets: To identify targets of the ‘Enhancer’ super-regions we used the EnhancerAtlas database (data downloaded in June 2016). This database associates its own set of enhancer positions with Ensembl transcripts for a set of 73 cell lines. For each ‘Enhancer’ super-region position from ENCODE overlapping a position from EnhancerAtlas in the same cell line, we associated the target provided by EnhancerAtlas with this ‘Enhancer.’ Remaining ‘Enhancers’ were characterized as having an unknown target.

iii) Gene body association: Each CpG assessed by RRBS, 850k or 450k was also intersected with LNCipedia and Ensembl transcript positions. The transcription start site (TSS) and transcription termination site (TTS) corresponding to each transcript were located on the genome. CpGs falling between a transcript-associated TSS and the corresponding TTS were described as ‘Gene body associated.’ If such a CpG was not already classified as ‘Promoter,’ ‘Enhancer,’ or ‘Dual,’ it was classified as ‘Gene body,’ while the remaining CpGs were assigned to the ‘Intergenic’ category. This annotation is available as a supplementary file on GEO (GSE198627_mbizet_GEO_Methylation850k_PLATFORMv2.1.txt.gz). The list of data source URLs is available in Table S2 (Additional File 3).

## Promoter and non-promoter regions

Analyses using only the ‘Promoter’ and ‘Non-promoter’ categories were carried out with the following grouping: ‘Promoter’ and ‘Dual’ cytosines were classified as ‘Promoter’ cytosines while ‘Enhancer,’ ‘Gene body,’ and ‘Intergenic’ cytosines were defined as ‘Non-promoter’ cytosines.

## Illumina default annotation

To compare the Illumina default annotation with our annotation, we collapsed the different levels of information provided by the Illumina 850k annotation file into the five categories used in our custom annotation. Associations with known transcripts were extracted from the ‘UCSC_RefGene_Group’ and ‘GencodeCompV12_Group’ columns. CpGs associated with ‘TSS 1500,’ ‘TSS 200,’ ‘1st Exon’ and ‘5’ UTR’ locations were categorized as ‘Promoter’ CpGs, while the remaining CpGs, associated with ‘Body’ and ‘3’ UTR’, were categorized as ‘Gene body’ CpGs. Enhancer-associated CpGs were retrieved from the ‘Phantom4_Enhancers,’ ‘Phantom5_Enhancers,’ and ‘450k_Enhancer’ columns. These CpGs were categorized as ‘Enhancer’ or ‘Dual’ CpGs, depending on their association with the ‘Promoter’ category. The remaining CpGs were categorized as ‘Intergenic.’

### Variance heterogeneity evaluation

Variance heterogeneity was assessed with a metric we called ‘variance heterogeneity measurement’ (*h*), which can be described as the distance between the mean standard deviation (representing the expected profile in a context of homogeneous variance) and a local regression model of the standard deviation along the methylation profile (reflecting the observed situation). It was computed as follows:

- The M value was computed for each probe with the formula described elsewhere [11].

- The standard deviation (*s_p_*) and the mean (*m_p_*) of the M-value of each probe across the HCT116 WT triplicates were computed so as to generate the vectors *s* and *m*.

- The observed profile was modelled with a local regression (loess) model fitting *s* as a function of *m* and a loess-smoothed value of the standard deviation was produced for each probe (s_p_*).

- In a context of homogeneous variance, *s* should be independent of *m*, so the expected profile should be modelled as a flat line at *s_0_*, the mean of *s.*

- A measurement of the variance heterogeneity (*h_p_*) was computed for each probe as the absolute difference between s_p_* (value from the observed model) and s_0_ (expected value in a homogeneous context).

- Finally, *h* was defined as the mean of all *h_p_* values.

### Differential methylation analysis

Differential methylation was assessed with a t-test applied to the M-values. In parallel, a delta beta value was computed as the absolute difference between the median beta value within each category. CpGs showing an adjusted p-value (Benjamini-Hochberg correction) <0.05 together with an absolute delta beta >0.2 were reported as differentially methylated.

## Supplementary Material

Supplemental Material

## Data Availability

Microarray data that support the findings of this study have been deposited in GEO with the accession codes GSE198628. All other data supporting the findings of this study are available from the corresponding author on reasonable request.
