# Supplementary material for: Improving Infinium MethylationEPIC data processing: re-annotation of enhancers and long noncoding RNA genes and benchmarking of normalization methods
Source: Epigenetics. 2022 Nov 10;17(13):2434–54. doi: 10.1080/15592294.2022.2135201 (PMC9665128; doi:10.1080/15592294.2022.2135201)
Supplement: Supplemental Material [file KEPI_A_2135201_SM2799.zip › supplement/Bizet et al_Revision_Additional File1_Supplementary Figures.pptx]

## Slide 1
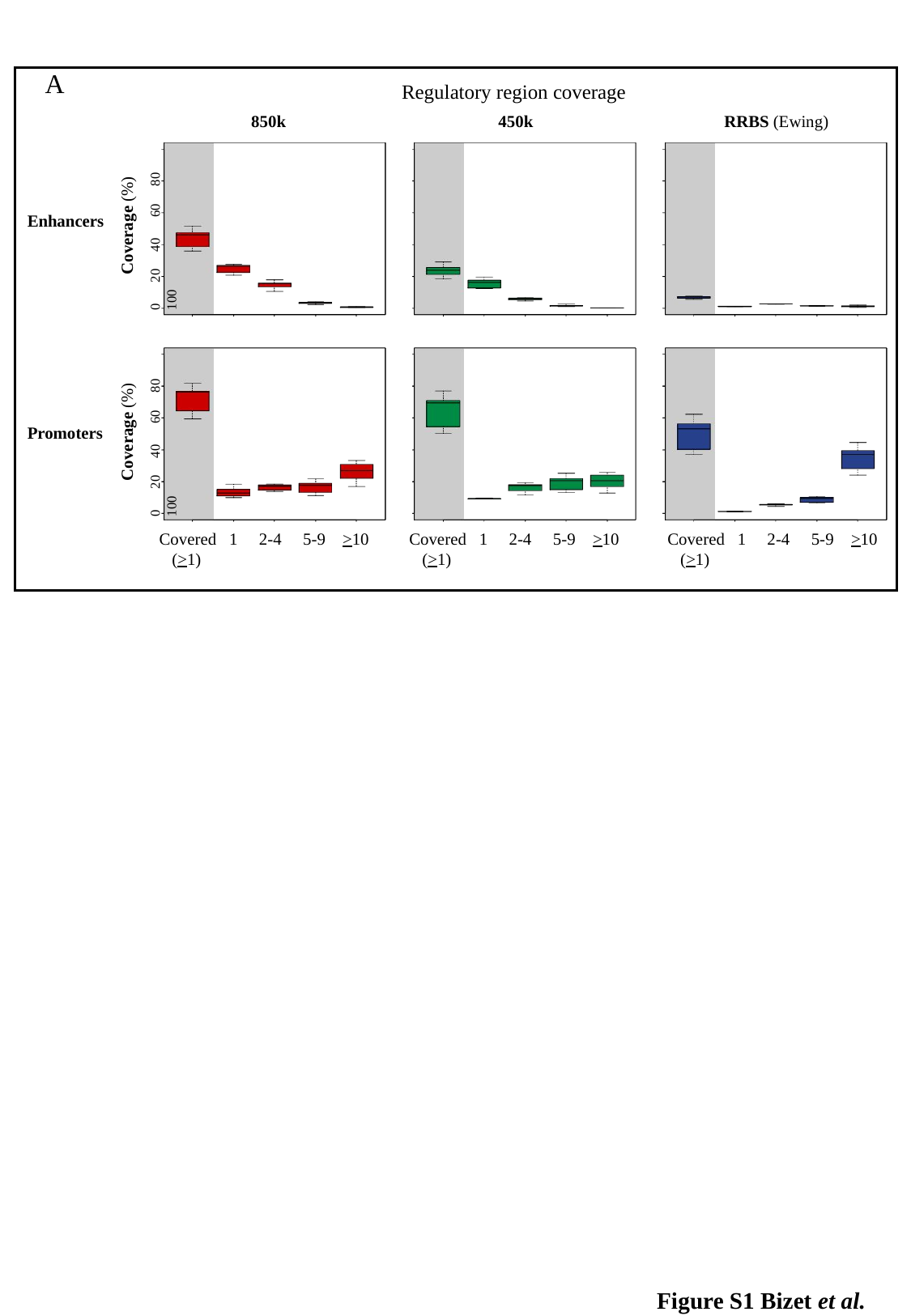

A
Regulatory region coverage
 850k	 450k	 RRBS (Ewing)
Coverage (%)
0 20 40 60 80 100
Enhancers
Coverage (%)
0 20 40 60 80 100
Promoters
 Covered 1 2-4 5-9 >10
 (>1)
 Covered 1 2-4 5-9 >10
 (>1)
 Covered 1 2-4 5-9 >10
 (>1)
Figure S1 Bizet et al.

## Slide 2
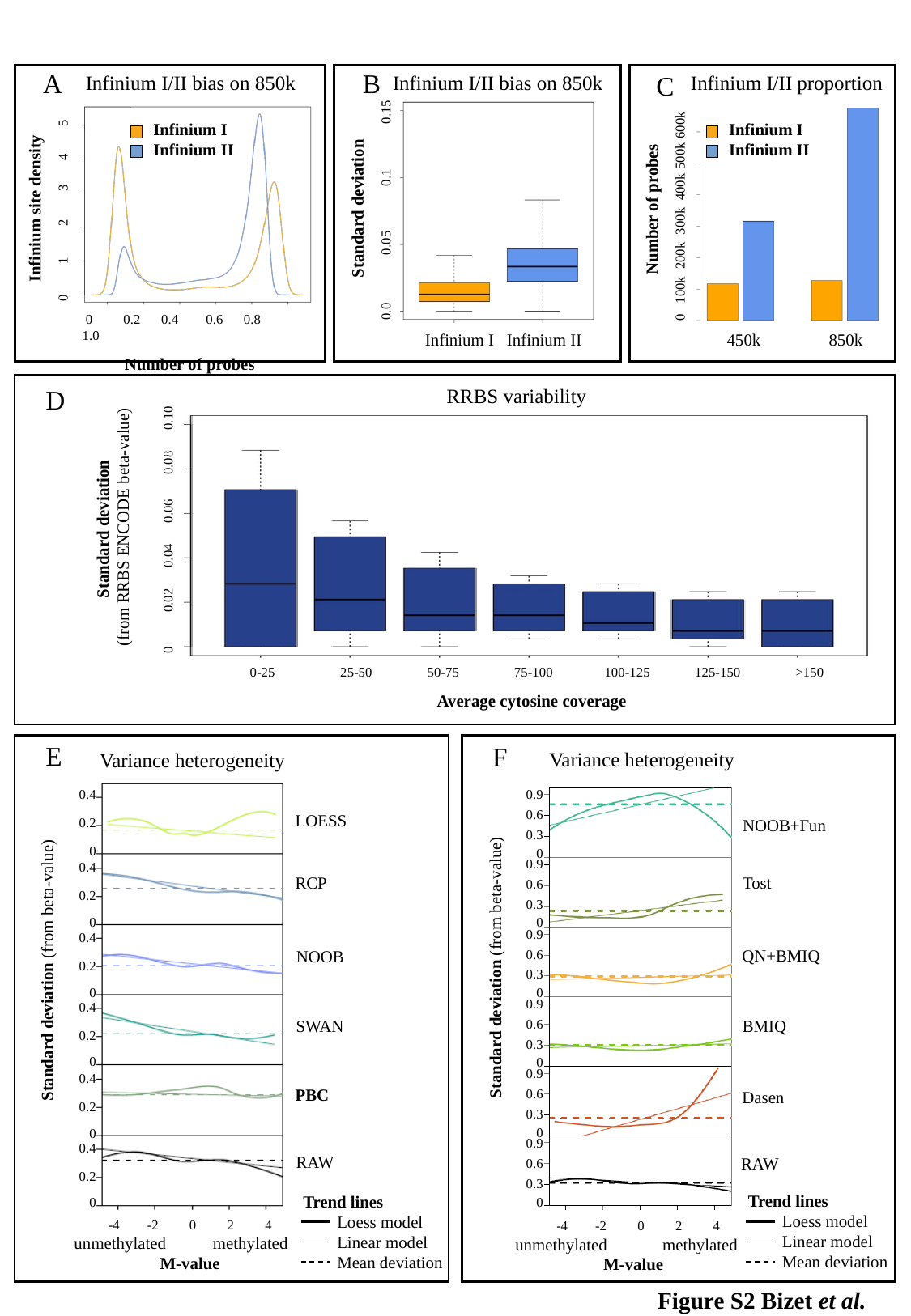

A
B
Infinium I/II bias on 850k
Infinium I/II bias on 850k
Infinium I/II proportion
C
Infinium I
Infinium II
Infinium I
Infinium II
Infinium site density
0 1 2 3 4 5
Standard deviation
0.0 0.05 0.1 0.15
Number of probes
0 100k 200k 300k 400k 500k 600k
 0 0.2 0.4 0.6 0.8 1.0
Number of probes
 Infinium I Infinium II
 450k 850k
RRBS variability
D
Standard deviation
(from RRBS ENCODE beta-value)
 0 0.02 0.04 0.06 0.08 0.10
 0-25 25-50 50-75 75-100 100-125 125-150 >150
Average cytosine coverage
E
F
Variance heterogeneity
Variance heterogeneity
0.4
 0.2
 0
0.9
 0.6
 0.3
 0
LOESS
NOOB+Fun
0.9
 0.6
 0.3
 0
0.4
 0.2
 0
RCP
Tost
0.9
 0.6
 0.3
 0
0.4
 0.2
 0
QN+BMIQ
NOOB
 Standard deviation (from beta-value)
 Standard deviation (from beta-value)
0.9
 0.6
 0.3
 0
0.4
 0.2
 0
BMIQ
SWAN
0.9
 0.6
 0.3
 0
0.4
 0.2
 0
PBC
Dasen
0.9
 0.6
 0.3
 0
0.4
 0.2
 0
RAW
RAW
Trend lines
 Loess model
 Linear model
 Mean deviation
Trend lines
 Loess model
 Linear model
 Mean deviation
 -4 -2 0 2 4
unmethylated methylated
M-value
 -4 -2 0 2 4
unmethylated methylated
M-value
Figure S2 Bizet et al.

## Slide 3
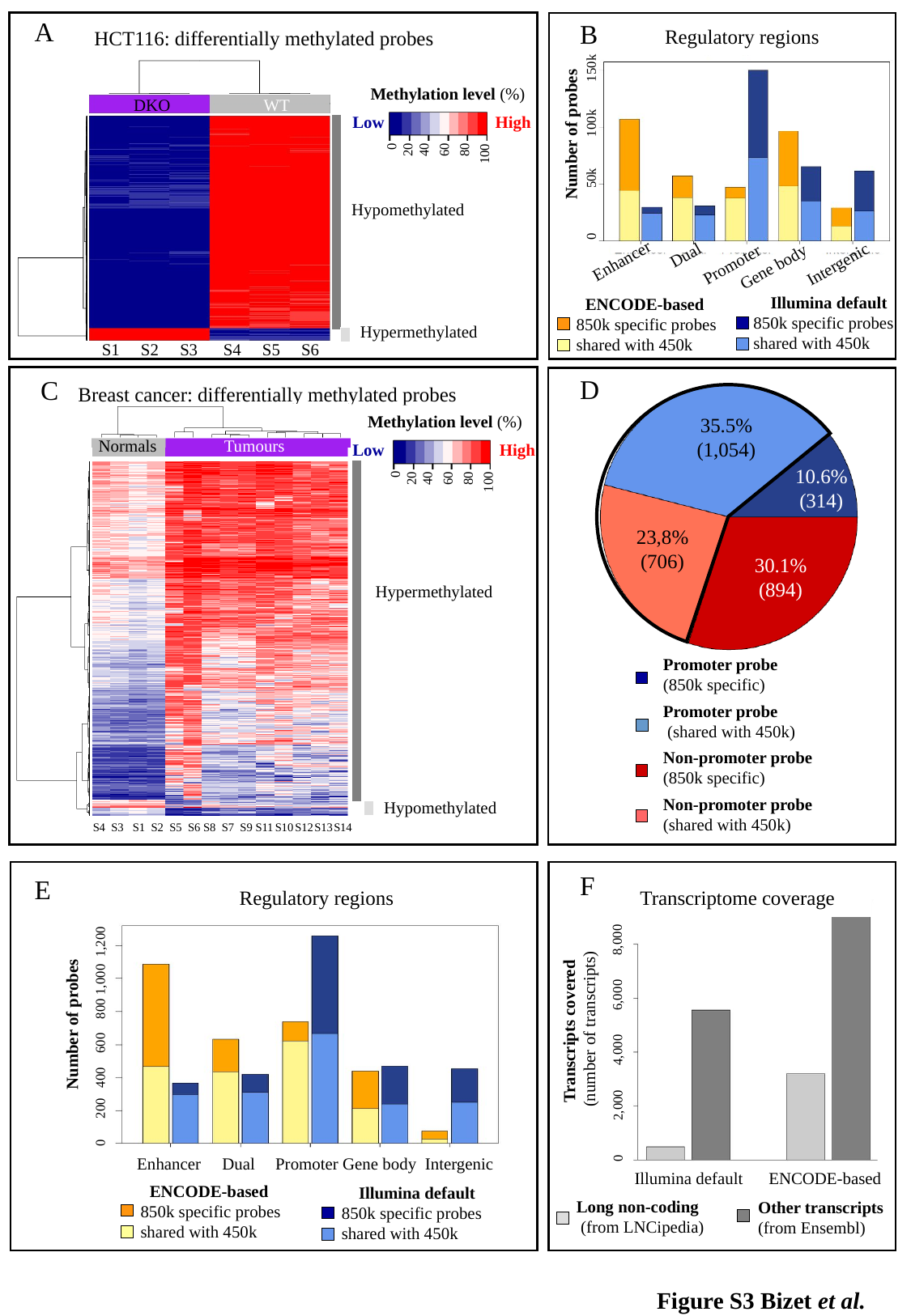

B
A
Regulatory regions
HCT116: differentially methylated probes
Methylation level (%)
0
20
40
60
80
100
 DKO WT
Low	 High
Number of probes
0 50k 100k 150k
Hypomethylated
 Hypermethylated
Dual
Enhancer
Intergenic
Promoter
Gene body
Illumina default
 850k specific probes
 shared with 450k
ENCODE-based
 850k specific probes
 shared with 450k
 S1 S2 S3 S4 S5 S6
D
C
Breast cancer: differentially methylated probes
Methylation level (%)
35.5%
(1,054)
0
20
40
60
80
100
 Normals	 Tumours
Low	 High
10.6%
(314)
23,8%
(706)
30.1%
(894)
Hypermethylated
 Hypomethylated
Promoter probe
(850k specific)
Promoter probe
 (shared with 450k)
Non-promoter probe
(850k specific)
Non-promoter probe
(shared with 450k)
 S4 S3 S1 S2 S5 S6 S8 S7 S9 S11 S10 S12 S13 S14
F
E
Regulatory regions
Transcriptome coverage
Transcripts covered
(number of transcripts)
0 2,000 4,000 6,000 8,000
Number of probes
0 200 400 600 800 1,000 1,200
Enhancer Dual	 Promoter Gene body Intergenic
 Illumina default	 ENCODE-based
ENCODE-based
 850k specific probes
 shared with 450k
Illumina default
 850k specific probes
 shared with 450k
Long non-coding
 (from LNCipedia)
Other transcripts
(from Ensembl)
Figure S3 Bizet et al.
