# Supplementary material for: Improving Infinium MethylationEPIC data processing: re-annotation of enhancers and long noncoding RNA genes and benchmarking of normalization methods
Source: Epigenetics. 2022 Nov 10;17(13):2434–54. doi: 10.1080/15592294.2022.2135201 (PMC9665128; doi:10.1080/15592294.2022.2135201)
Supplement: Supplemental Material [file KEPI_A_2135201_SM2799.zip › supplement/supplementary legends.docx]

**Additional Files**

***Additional File 1 (.pdf): Supplementary figures***

***Fig. S1 (associated to Figure 2) Coverage of the 850k bead chip as compared to 450k and RRBS:*** Proportion of regulatory regions (enhancers *top*, promoters *bottom*) covered by (from *left* to *right* in each plot) at least one, exactly one, two to four, five to nine, or more than ten targeted cytosines with 850k (*red*), 450k (*green*), and RRBS (*blue*). Each boxplot represents the distribution of the coverage among the nine cell lines as provided by ENCODE. The region in *grey* in each plot is highlighted in Figure 1D.

***Fig. S2 (associated to Figure 3) Evaluation of methods correcting for 850k bias:*** (A) Density profile of raw 850k probes as a function of the beta-value. Profiles for Infinium I and II probes are shown, respectively, in *orange* and *medium* *blue*. Infinium II probes display a lower dynamic range than Infinium I probes. (B) Boxplots showing the distribution of the standard deviation obtained upon cytosine methylation level assessment with triplicate 850k arrays for Infinium I (*orange*) and Infinium II (*medium blue*) measurements. (C) Barplot of the number of cytosines covered by Infinium I (*orange*) and Infinium II (*mid-blue*) probes in 450k (*left*) and 850k (*right*) arrays. (D) RRBS between-replicate variability. Boxplots show the distribution of the standard deviation of the DNA methylation level between duplicate HCT116 cell samples, for cytosines assessed with an increasing read average. (E-F) Variance heterogeneity profiles of raw 850k data (*black*) and normalized data. The average methylation value is shown on the x-axis and the standard deviation on the y-axis.. The dashed line indicates the mean variance (*i.e.* s0 parameter for variance heterogeneity measurement computation). The thick curve is the loess model of the variance as a function of the average methylation level (*i.e.* the profile of the sp* parameter for variance heterogeneity measurement computation). A flat profile would indicate variance homogeneity. The thin line is the linear model of the variance as a function of the average methylation level, its slope was used to determine if normalization was increasing variance at unmethylated (E; negative slope) or methylated (F; positive slope) side. The normalisation method is specified in the right side of the plot. Bold indicated a method leading to less variance heterogeneity than raw data. RAW: raw Infinium data; PBC: peak-based correction from the wateRmelon package; NOOB: Normal exponential convolution using out-of-bounds; SWAN: Subset quantile Within-Array Normalisation from the minfi package; QN+BMIQ: pipeline formed by quantile normalisation on intensities followed by beta-mixture quantile normalisation; LOESS: local-regression between-array normalisation; RCP: regression on correlated probes method; Tost: categorical SQN from the Touleimat and Tost pipeline; NOOB+Fun: pipeline composed of NOOB correction followed by functional normalisation; Dasen: Dasen pipeline from the wateRmelon package.

***Fig. S3 (associated to Figure 4) Differential methylation analysis of HCT116 WT vs HCT116 DKO cells:*** (A) Heatmap of the 10,000 most highly differentially methylated probes identified on the 850k array upon comparing HCT116 DKO (samples S1 to S3, *purple* rectangle) and HCT116 WT cells (samples S1 to S3, *grey* rectangle) after PBC normalization. The methylation level is represented on a *blue* (unmethylated) to *red* (methylated) scale. Hypomethylated probes are highlighted by a *dark grey* vertical bar. (B) Barplot of the number of differentially methylated cytosines that can be assessed exclusively by 850k (*dark*) or that are common to 850k and 450k (*light*). The differentially methylated cytosines are associated with ‘Enhancer’, ‘Dual’, ‘Promoter’, ‘Gene body’, and ‘Intergenic’ regions according to the ENCODE-based (*orange*) or Illumina default annotation (*blue*). (C) Heatmap of the differentially methylated probes identified on the 850k array upon comparing breast tumour samples (samples S5 to S14, *purple* rectangle) with normal breast tissue samples (samples S1 to S4, *grey* rectangle) after BMIQ normalization. The methylation level is represented on a *blue* (unmethylated) to *red* (methylated) scale. Hypermethylated and hypomethylated probes are highlighted respectively a *dark grey* and a *light grey* vertical bar. (D) Pie chart of the proportion of differentially methylated promoter and non-promoter probes. *Blue*: promoter, *red*: non-promoter, *light*: probes common to the 850k and 450k versions, *dark*: probes specific to the 850k array. The part surrounded in black contains probes common to the two versions. (E) Barplot of the number of differentially methylated cytosines that can be assessed exclusively by 850k (*dark*) or that are common to 850k and 450k (*light*). The differentially methylated cytosines are associated with ‘Enhancer’, ‘Dual’, ‘Promoter’, ‘Gene body’, and ‘Intergenic’ regions according to the ENCODE-based (*orange*) or Illumina default annotation (*blue*). The ENCODE-based annotation shows a lower proportion of differentially methylated cytosines not associated with any feature (*i.e.* ‘Intergenic’). This leads to better interpretability of results. (F) Barplot showing the number of transcripts associated with at least one differentially methylated cytosine, according to the reference annotation (Illumina default *left*, ENCODE-based *right*) and the transcriptomic database (LNCipedia *light grey*, Ensembl *dark grey*). It highlights strong improvement in the number of transcripts identified with the ENCODE-based annotation as compared to Illumina’s default annotation even when BMIQ is used for normalization. WT: wild type; DKO: double knock-out for DNMT1 and DNMT3

***Additional File 2 (.xls): List of Promoter/Enhancer-transcript associations***

Table describing the transcript type for each transcript from Ensembl and LNCipedia. Possible types are Coding, small noncoding (small-NC) or lncRNA. In case of lncRNA three extra-types are possible depending on the TSS location: ‘lncRNA (promoter)’ when the TSS is in a promoter region, ‘eRNA’ when TSS is in an enhancer region and ‘lncRNA (unknown)’ if the TSS is located in promoter or enhancer depending on the cell line or if the information is not available.

***Additional File 3 (.xls): List of data source URLs***

Table listing the public data files used for the 850k reannotation.
